# Sex and gender in medical education: a national student survey

**DOI:** 10.1186/s13293-016-0094-6

**Published:** 2016-10-14

**Authors:** Marjorie R. Jenkins, Alyssa Herrmann, Amanda Tashjian, Tina Ramineni, Rithika Ramakrishnan, Donna Raef, Tracy Rokas, John Shatzer

**Affiliations:** 1 Texas Tech University Health Sciences Center, Laura W. Bush Institute for Women’s Health, 1400 Coulter Ave, Amarillo, TX 79106 USA; 2 Albany Medical College, Albany, NY USA; 3 Belmont University, Nashville, TN USA; 4 Johns Hopkins University, Baltimore, MD USA

## Abstract

**Background:**

Gender- and sex-specific medicine is defined as the practice of medicine based on the understanding that biology (dictated by sex chromosomes) and social roles (gender) are important in and have implications for prevention, screening, diagnosis, and treatment in men and women. In light of the many ways that sex and gender influence disease presentation and patient management, there have been various initiatives to improve the integration of these topics into medical education curriculum. Although certain schools may include the topics, their impact on the student body’s knowledge has not been as fully studied. By studying the opinions of US allopathic and osteopathic-enrolled students on the extent to which their schools address these topics and their understanding of these topics, this study examined the role of gender specific medicine in the US medical school curriculum.

**Methods:**

An email solicitation with link to an anonymous survey was sent to approximately 35,876 student members of five US medical student organizations. The survey instrument consisted of yes/no, multiple choice, and attitude awareness questions. Data was analyzed as a complete data set to evaluate national trends and via subset analysis using chi-square, paired *t* test, and one-way anova.

**Results:**

A total of 1097 students responded. The majority of respondents strongly agreed that sex and gender medicine (SGBM) improves patient management (96.0 %) and should be included as a part of the medical school curriculum (94.4 %). Only 2.4 % of participants agreed that SGBM is the same as Women’s Health. When asked specifically about inclusion of an identified sex and gender-based medicine curriculum at their institution, students answered not sure at 40.8, 25.1, 19.1, and 20.3 % from first year to fourth year, respectively. Males reported a higher rate of exposure to SGBM content areas (in medical history taking, domestic violence) than women.

**Conclusions:**

Medical students recognize the differentiation between SGBM principles and women’s health, and understand the translational value of sex and gender-specific principles in the clinical setting. However, current curricular offerings fall short of providing students with adequate coverage of specific evidence-based health differences.

**Electronic supplementary material:**

The online version of this article (doi:10.1186/s13293-016-0094-6) contains supplementary material, which is available to authorized users.

## Background

Beginning in the 1960s, as disparities in women’s rights came to the fore-front, the medical field began to develop an increased interest in women’s health [1]. In a traditional sense, the field of women’s health focused on those topics related to pregnancy and reproduction almost exclusively. However, following the publication of the Institute of Medicine’s report, *Does Sex Matter* [2], in 2001 and the publication of *Principles of Gender-Specific Medicine* in 2004, an increasing emphasis was placed on the importance of sex and gender medicine [3].

While sex is defined as the unique physical makeup of men and women due to chromosomal, reproductive, and hormonal differences, gender focuses on the implications of society’s psychosocial framework relating to the norms of being a man versus a woman [4]. Therefore, sex and gender medicine is defined as understanding that biology and social roles are important in and have implications for prevention, screening, diagnosis, and treatment [5].

Because sex and gender play a significant role in the medical management of patients, it is paramount that these topics be incorporated into medical research and education. These differences in physiology and pathophysiology between males and females are evident in many disease processes, diagnostic tests, and treatment options. In a recent review of scientific literature, over 3000 articles dealing with sex and gender differences in disease presentation were found [3]. For instance, it is widely accepted that cardiovascular disease (CVD) presents differently in men and women, and that the knowledge of such differences is a critical component of the clinical armamentarium [6]. In addition to disease presentation, sex and gender influence treatment efficacy. At the most fundamental level, one’s sex and gender will affect pharmacokinetics and pharmacodynamics. For example, in the case of the sleep aid, zolpidem, women have a much lower metabolism rate resulting in higher plasma concentrations and sensitivity [7]. Thus, at equal doses, zolpidem causes longer periods of impairment in women. In February 2014, zolpidem became the first drug to have different US Food and Drug Administration (FDA) guidelines for dosing in men versus women [8].

Acknowledging that sex and gender have medical implications has led to various initiatives to improve the integration of these topics into the medical education curricula. In 1994 and 1995, the Association of American Medical Colleges (AAMC), in response to a Congressional request, surveyed the inclusion of women’s health and sex and gender medicine in approximately 100 medical schools [9, 10]. Following the AAMC surveys, four additional studies between 1997 and 2002 were conducted to track curricular progress in incorporating this topic. These studies identify a lack of comprehensive integration of sex and gender medicine into the basic sciences and clinical education [11, 12].

In 2011, *A Sex and Gender Based Medicine Faculty Survey* was administered to faculty from the majority of medical schools in the USA and Canada. Of the 44 schools that responded, 70 % indicated that they did not have a formal sex- and gender-specific integrated medical curriculum. When asked if adequate coverage was granted to ten specific health topics in which sex- and gender-based evidence exists, 45–70 % ranked their coverage as minimal [13].

### National student survey

Based on these previous studies, it is apparent that medical education in the USA has not adequately integrated sex and gender-based medicine into its curricula. Thus, the purpose of this study was to determine, from the perspective of medical students, how effectively medical schools integrate evidence-based sex and gender differences into curricula, as well as these students’ knowledge, attitudes and awareness of sex and gender medicine. This study represents the first national survey of medical students’ knowledge, attitudes, and awareness of sex and gender.

## Methods

### Sample

A convenience sample of male and female US medical student members of the following national medical student organizations: American Medical Student Association, the American Medical Women’s Association, the Asian Pacific American Medical Student Association, the Latino Medical Student Association, and the Student National Medical Association was studied. Inclusion criteria were students who attended 4-year MD or DO medical schools where either all 4-year or the last two clinical years were completed on campuses located in the USA. Students from international medical schools and naturopathic medical schools were excluded.

A total of approximately 35,876 medical student members of the organizations received the solicitation email and 1097 responded. The estimated total membership of the organizations is likely a duplicate count as cross membership with other organizations is possible. However, the achieved sample size (1097) ensures a 99 % confidence level with 4 % margin of error.

### Instrumentation

The online survey contained seven topic areas: demographics, attitudes and awareness, women’s health, men’s health, gender-specific medicine, content areas and specific curricular items. Questions consisted of yes/no, multiple choice, scaled, and free-text entry. For the purpose of the survey, the following definitions of “women’s health”, “men’s health”, and “gender-/sex-specific medicine” were operationalized as follows:

*Women’s Health: Screening, diagnosis and management of conditions unique to female anatomy, more prevalent in women, and/or more consequential in women*

*Men’s Health: Screening, diagnosis and management of conditions unique to male anatomy, more prevalent in men, and/or more consequential in men*

*Sex and Gender-Specific Medicine: The practice of medicine based on the understanding that biology (dictated by sex chromosomes) and social roles (gender) are important in and have implications for prevention, screening, diagnosis, and treatment; and in the design and implementation of health research, policy, programs and services in men and women.*

Demographic data s included: student’s age, school type, program, year in school, and field of study prior to medical school. The attitude and awareness component used a five-point Likert Scale to assess perceptions of sex and gender medicine’s importance and respondent’s familiarity with the topic (1 = strongly disagree; 2 = disagree; 3 = neither agree nor disagree; 4 = agree; 5 = strongly agree). Within the women’s health, men’s health and gender-specific medicine sections, respondents were asked to indicate whether their institution included specific curricular offerings such as required reading, objective structured clinical exams (OSCEs), fellowships or residency programs in these respective areas. The survey included these questions regarding institutions’ educational resources and training programs in women’s health, men’s health, and sex- and gender-specific health to better assess integration of these areas.

In the content areas subsection, students indicated the extent to which their institution covered sex and gender differences in major content areas such as pulmonology and endocrinology via a four-point Likert scale (1 = no course or lectures, 2 = minimal coverage, 3 = moderate coverage, 4 = extensive coverage) that included definitions for minimal, moderate and extensive coverage. The specific curricular items subsection included yes/no questions regarding whether the student’s medical education to date included curricular opportunities pertaining to certain evidence-based health differences between men and women. The content areas were chosen as having level one or two evidence for the differences described. Although free-text entry was included in the survey, the responses and comments were not analyzed in this manuscript. Qualtrics survey software was used to build and administer the survey and all data were automatically de-identified through the software; questions in the demographics subsection did not include any identifying information. Because the email solicitation was sent to student members at different levels in their medical education, survey logic was incorporated to ensure that questions remained relevant to the student based upon their current education and experience. For example, only third and fourth year students were shown questions regarding specific institutional offerings (Question 6a, 7a) as a majority of medical school program introduce electives during those years (Additional file 1). The initial survey was created by a group of faculty and students at Texas Tech University Health Sciences Center. For the purposes of this work, extensive revisions were incorporated by the authors, medical students at Albany Medical College, and a survey design and statistical analyst from Belmont University. Once drafted, the survey was beta tested by seventeen students from Texas Tech Health Science Center and Albany Medical College in addition to national sex and gender medicine faculty experts from the Sex and Gender Women’s Health Collaborative (http://sgwhc.org/).

### Procedure

The Gender-Specific and Men’s and Women’s Health Curriculum National Survey was conducted under an IRB-approved protocol through Texas Tech University Health Sciences Center, and all participants were informed in the email solicitation that, by completing the de-identified survey, they were voluntarily consenting to participate in a research study. Participants were recruited through third-party distribution of an IRB approved email specifically concerning the survey. Student members of the five participating medical student organizations were contacted by their respective organization. As part of their membership, students of these organizations had previously agreed to be contacted for various purposes including surveys. No compensation or incentive was offered to study subjects and participation was strictly voluntary. The email solicitation described the intent of the study and included the link to the electronic survey. The survey required approximately 10–15 min to complete. The link was active for 110 days, and a reminder email was sent out by the organizations before the link expired.

### Statistical analysis

Data from the de-identified survey responses were analyzed both as a complete data set to evaluate national trends and via subset analysis according to demographic data collected. Gender differences between mean responses to Likert scale questions were analyzed by conducting independent samples *t* tests, and mean differences by year-in-school were analyzed by performing one-way ANOVAs and means for homogenous subsets were further explored and validated through the use of Tukey post hoc tests. ANOVA analysis was also performed to compare exposure to sex and gender differences between different class years. For Likert scale questions, care was taken to set any non-responses of “don’t know” or “not sure” to system missing before generating or testing means.

Binary data collected in response to yes/no questions and other nominal data collected through yes/no questions with additional response options were analyzed through the use of Pearson’s chi-square tests. Subsequent to the analysis of mean differences, for the purpose of reporting respondents’ attitudes and perceptions in clearer fashion, questions were collapsed into categories of agree (containing response of “strongly agree” and “agree”), and disagree (“strongly agree” and “disagree”). In order to address gender bias in response rate, weighted responses were calculated based on data from the American Association of Medical Colleges’ total enrollment records from 2015–2016 divided by sex.

## Results

### Demographics

One hundred and fifty four schools were represented by the survey respondents, with participation from 1191 students, 1097 of which met the inclusion criteria for final analysis. The highest frequency of respondents attended medical school in New York (11.2 %), followed by Pennsylvania (6.3 %) and Missouri (5.8 %). Students from multi-disciplinary medical schools (those affiliated with other health science programs such as pharmacy and nursing schools) comprised 81.4 % of respondents. The respondents were divided almost equally between those attending state-supported public institutions (45.7 %) and those attending private institutions (49.2 %). Medical schools that focused on clinical care and community engagement had the most student responses (Table 1). Students from MD only schools composed 66.9 % of respondents, as opposed to DO or combined MD programs.

**Table 1:** Student and school demographics

Age	<20	21–35	26–30	31–35	>35
% of respondents	0.9	50.7	37.4	7.5	3.4
Gender	Female	Male	Other		
% of respondents	74.3	25.2	0.5		
School year	MS1	MS2	MS3	MS4	5+ years
% of respondents	25.4	33.7	21.5	17.6	1.8
Primary focus of school	Clinical care	Community engagement	Research	Medical education	Other
% of respondents	73.0	43.6	38.1	75.3	1.8
Program type	MD only	MD/PhD or other degree program	DO only	DO/PhD or other degree program	Other
% of respondents	66.9	10.0	21.1	1.6	0.5

As reported by student, multi-response question, *n* = 1097

Of all respondents, 25.4 % were in their first year of medical school, 33.7 % in their second year, 21.5 % in their third year, 17.6 % in their fourth year, and 1.8 % were in their fifth year or more. The majority (88.1 %) of respondents were between the ages of 21 and 30. A significant portion of the respondents reported the biological sciences to be their prior field of study in undergraduate (69.0 %). In terms of gender distribution, 74.3 % of respondents were women and 25.2 % were men (Table 1).

### Attitudes and perceptions

With respect to attitudes and perceptions regarding sex and gender medicine, 85.5 % of respondents reported to be familiar with sex and gender differences in medicine. Only 2.1 % of respondents believed sex and gender medicine to be the same as women’s health, and less than one third (31.0 %) agreed with the statement that women’s health focuses solely on issues specific to females, such as menarche, pregnancy, and menopause. The majority of students also agreed that content in their curriculum is primarily related to males (63.2 %). And, nearly all respondents agreed that knowing sex and gender medicine improves one’s ability to manage patients and should be included as a part of the medical school curriculum (96.0 and 94.2 %, respectively) (Table 2).

**Table 2:** Medical student attitudes and perceptions of sex- and gender-based medicine, question 5

	I am familiar with the topic of sex and gender differences in medicine	Women’s Health focuses solely on issues specific to females (menarche, pregnancy, menopause)	Sex and gender medicine is the same as women’s health	Knowing sex and gender differences improves one’s ability to manage patients	The majority of medical knowledge is based on data obtained from males	Medical education should include the teaching of sex and gender differences	My medical education has included the teaching of sex and gender differences
Year in medical school	Percentage agree/strongly agree						
First year	79.3	31.2	4.0	97.8	58.3	93.8	48.6
Second year	86.4	29.6	1.6	96.2	63.9	94.8	62.8
Third year	87.1	31.1	1.3	94.0	63.8	93.2	57.4
Fourth year	89.1	33.3	1.6	95.3	63.1	94.8	66.7
Average	85.3	31.0	2.1	96.0	63.2	94.2	58.6
ANOVA (*p* value)	0.004	0.871	0.033	0.004	0.678	0.050	0.001
ANOVA *F*	4.527	0.236	2.914	4.533	0.506	2.615	5.246

Students were asked their level of agreement or disagreement via four-point Likert scale about certain statements regarding sex and gender education in medical education, from “strongly disagree” to “strongly agree”. (*n* = 1070)

One-way ANOVA tests revealed that class years differed significantly in their attitudes and perceptions of sex and gender medicine (Table 2). In particular, differences were observed between first and, to a lesser degree, second year students and students in their third and fourth years. Perceived exposure with the topic of sex and gender medicine grew significantly as class year progressed, and the perception that sex and gender medicine is the same as women’s health decreased. However, opinions on whether knowing sex and gender differences improves ability to manage patients remained at 94 % or greater across the four years (first year: 97.8 %; second year: 96.2 %; third year: 94.0 %; fourth year 95.3 %) (Table 2).

### Sex- and gender-based medicine curricular offerings

Despite the fact that more than half of respondents reported to be familiar with the topic, when asked about the inclusion of an identified sex and gender-based curriculum in their medical education, only 31.1 % of students responded yes (42.0 % responded no, and 26.9 % were unsure; *n* = 1096). When asked if the curriculum included specific classes or programs on sex and gender differences, 31.6 % of respondents chose no, and 48.1 % of respondents chose yes. Only 43.1 % of students report that their curriculum has given them a better understanding of sex and gender medicine, and only 34.5 % report they would feel prepared to manage sex and gender difference in healthcare.

Classes responded differently to whether their curricula have provided a better understanding of sex and gender medicine as well as whether their curricula included the teaching of sex and gender differences. Specifically, second year and fourth year students were more likely than first and third year students to report their curricula included sex and gender education (first year = 31.0 %; second year = 54.2 %; third year = 50.6 %; fourth year = 57.8 %) and agree that their curricula has given them a better understanding of sex and gender medicine (first year = 28.7 %; second year = 48.9 %; third year = 43.8 %; fourth year = 50.3 %).

Students were asked to report the extent of coverage of sex- and gender-based medicine topics within ten broad fields of medical study where noted sex and gender differences exist: domestic violence, substance abuse, mental health, nutrition, pharmacology, pulmonology, cardiology, rheumatology, infectious disease, and endocrinology. Moderate to extensive coverage was reported for endocrinology (76.4 %), medical history taking (72.5 %), mental health (66.2 %), rheumatology (64.1 %) and cardiology (64.0 %), infectious disease (59.4 %), pharmacology (59.1 %), substance abuse (57.0 %), domestic violence (56.3 %), pulmonology (54.7 %), and nutrition (45.2 %).

### Sex- and gender-based medicine offerings versus knowledge

Students were also asked whether select examples of specific evidence-based health differences between men and women—(a) presenting symptoms of myocardial infarction (MI), (b) outcomes after low impact fractures (c) dosing of zolpidem, and (d) narcotic addiction—had been included in their medical education to date. The selected examples were included because significant differences in outcomes for males and females are well-documented in research over the past decade, yet is unknown if this evidence has been incorporated into training.

Reported Sex and Gender Based Medicine (SGBM) coverage (question 10) was then compared to knowledge of specific SGBM examples (question 11) to elucidate disparities in the perception of coverage versus factual knowledge. When comparing exposure to sex and gender differences between different class years, results showed significant differences between class years in exposure to specific health topics, including domestic violence, mental health, nutrition, pharmacology, pulmonology, cardiology, infectious disease, and endocrinology (Table 3). Reported exposure to sex and gender differences in presentation of symptoms of myocardial infarction, use of aspirin for the prevention of MI and stroke, and victims of domestic violence displayed a trend of increasing with class year, whereas fewer fourth year students reported exposure to sex and gender differences in dosing of zolpidem, narcotic addiction, and smoking cessation than second or third year students (Table 4).

**Table 3:** Student awareness of topics in sex and gender medicine, question 10

	Content area										
	Medical history taking *n* = 1025	Domestic violence *n* = 975	Substance abuse *n* = 963	Mental health *n* = 968	Nutrition *n* = 969	Pharmacology *n* = 957	Pulmonology *n* = 915	Cardiology *n* = 927	Rheumatology *n* = 879	Infectious disease *n* = 920	Endocrinology *n* = 911
Year in medical school	Percentage indicating “Moderate to Extensive” or “Extensive Coverage” of sex and gender differences in each topic										
First year	69.7	47.7	57.8	59.3	60.3	69.8	70.9	72.9	70.5	68.4	84.1
Second year	75.8	53.2	53.4	64.5	45.9	56.2	50.0	60.4	64.1	57.5	79.4
Third year	74.8	61.4	58.7	69.1	41.0	59.8	53.8	66.4	63.9	61.6	72.2
Fourth year	67.0	65.6	60.2	73.4	32.8	53.2	48.9	58.9	58.5	52.2	69.8
Average	72.5	56.5	56.8	66.3	45.3	59.2	54.7	64.0	64.0	59.5	76.5
ANOVA (*p* value)	0.190	0.000	0.413	0.007	0.000	0.001	0.000	0.012	0.073	0.003	0.010
ANOVA *F*	1.589	6.745	0.956	4.060	9.138	5.843	10.599	3.660	2.334	4.801	3.818

**Table 4:** Inclusion of evidence-based health differences in medical education, question 11

	Evidenced-based health differences between men and women					
	Presenting symptoms of MI	Using aspirin for prevention of MI and stroke	Dosing of zolpidem	Narcotic addiction	Smoking cessation	Victims of domestic violence
Year in medical school	Percentage answering “Yes”					
First year	63.8	38.9	7.7	20.2	25.2	38.2
Second year	86.7	46.9	13.7	31.4	34.2	61.8
Third year	92.9	54.3	16.0	33.3	36.8	77.2
Fourth year	94.2	60.7	13.6	26.7	28.3	80.1
Average	83.4	49.0	12.7	28.1	31.4	62.6
Chi-square (*p* value)	0.000	0.000	0.033	0.004	0.019	0.000

Students were asked to answer “yes” or “no” regarding whether their medical education to date had included evidence-based health differences between men and women in regards to the topics listed

## Discussion

Results suggest that a majority of US medical students are familiar with the topic of sex and gender medicine as a separate entity from women’s and men’s health, and believe this topic will improve their abilities to treat patients in the future. However, when asked if they feel as though their curriculum has prepared them to manage these differences at a clinical level, less than half of students agreed. When asked specifically about inclusion of an identified sex and gender-based medicine curriculum at their institution (question 8a), students answered not sure at 40.8, 25.1, 19.1, and 20.3 % from first year to fourth year, respectively. It is important to note that students answered “no” to the same statement (question 8a) at rates of 35.7, 40.1, 49.8, and 42.3 % from first year to fourth year, respectively. Given that upper level students have had a broader exposure to the institution’s entire curriculum, this suggests a gap in existing medical school curricula with respects to sex- and gender-based exposure and that a basic review of the definitions of sex and gender is merited.

**Fig. 1:**
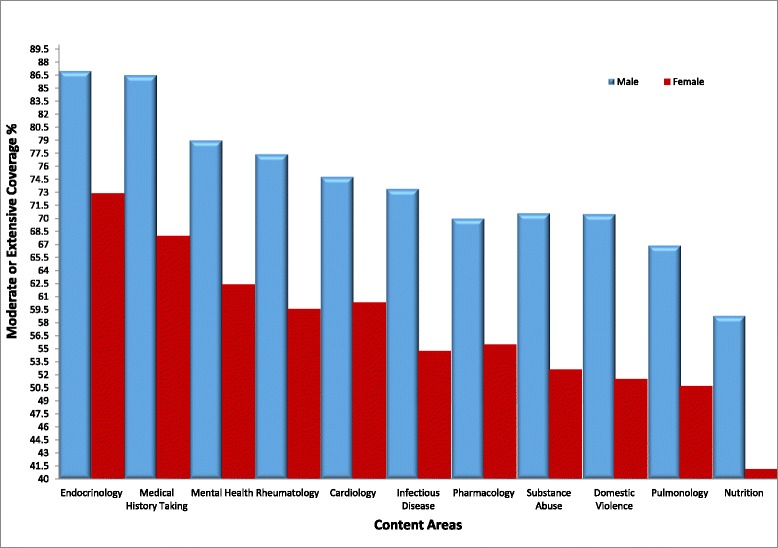
Student perception of coverage of sex and gender differences within content areas. Students’ perceptions of the extent of coverage of sex- and gender-based medicine in their current curriculum, categorized by topic and gender. Students were asked on a four-point Likert scale the extent to which their institutions cover sex and gender differences in specific fields, from no coverage to extensive coverage

The majority of respondents were female; however, male students consistently perceived coverage to be “moderate to extensive” at greater percentages than female students (Fig. 1). In addition, when asked if their curriculum had prepared them to manage sex and gender differences in healthcare or given them a better understanding of sex and gender differences, males were more likely to agree (55.1 %) than females (38.9 %). Survey studies have shown males to be more likely to express confidence in their response than females, even though females were found to be more accurate in their response despite their lower confidence [14]. Additionally, in a study where males and females were asked to answer sex and gender specific test questions, males were less likely than females to answer female specific questions correctly. However, females correctly answered male-specific questions at the same frequency as males [15]. Taken together, these differences raise an interesting question about how gender influences perception and learning. It may be possible that the male student’s increased confidence as described by Theobald et al. (2015) drives the tendency to assume that topics have been covered or mastered when in fact they were not. It also may be possible that the traditional male model in which medicine is taught may skew female students to notice more inconsistencies as they pertain to themselves. Either way, it demonstrates the importance of improving the understanding of sex and gender medicine as it impacts the health of both men and women. There are many instances where diseases are assumed to be “female-” and male-specific presentations are not adequately taught.

A significant international body of research on sex and gender differences in various pathologies continues to grow, yet it appears to be inconsistently applied to clinical practice. When asked about the inclusion of sex and gender differences in specific curricular topics such as cardiology, pharmacology, substance abuse, and rheumatologic disease, the perception of “moderate to extensive” coverage ranged from 52–76 %. There was discordance between expressed knowledge and perceived amount of exposure to specific evidence-based health differences in certain topics such as dosing of zolpidem and narcotic addiction (Table 5). However, based on the analysis across topics, students who reported knowledge of specific examples of sex and gender differences in cardiology and rheumatology also reported to have had statistically greater coverage in that topic material under content areas (Table 5). Certain sex differences, such as female- and male-specific presentation and treatment of cardiovascular disease have been more extensively researched and publicized during recent years. As a result, it has become a better understood topic in sex and gender medicine and nearly 90 % of student respondents reported coverage of sex and gender differences in presenting symptoms of myocardial infarction. The successful integration of sex and gender as it pertains to cardiovascular health supports the argument that other topics could also be successfully incorporated into the main curriculum.

**Table 5:** Reported extent of coverage of sex and gender content area versus awareness of specific sex- and gender-based health differences within content area

Content area	Percent reporting “Moderate to Extensive” sex and gender coverage^a^	Question 11. “There are evidenced-based health differences between men and women in regard to the topics listed”	Percent reporting “Moderate to Extensive Coverage” and Answering “Yes” to question 11	Sig. (two-tailed)	Mean difference
Cardiology	64.0	Presenting symptoms of myocardial infarction	56.9	0.000	0.401
Rheumatology	64.1	Outcomes after low impact fractures in adults	47.5	0.000	0.370
Substance abuse	57.0	Narcotic addiction	29.7	0.000	0.672
Pharmacology	59.1	Dosing of zolpidem	13.1	0.000	0.591

^a^Question 10. sex- and gender-specific content areas, “For each of the following topics discussed please indicate the extent to which your institution covers sex and gender differences in your curriculum”

The initiative to embed sex and gender education into medical training has begun to take hold at the graduate level in fields such as emergency medicine [16]. Resident electives and fellowships in sex- and gender-based medicine are established as a way to better serve the patients seen in the emergency room [8]. These electives and fellowships at the graduate training level validate a direct impact on patient care. In addition to introducing concepts of sex and gender medicine during residency and fellowship, its incorporation throughout medical education will allow all students to gain proficiency in the topic and develop a unique framework from the onset of the student’s clinical years.

### Limitations

While there is evidence that respondents can be more detailed in their responses in a shorter period of time when taking a survey online, there are known logistic difficulties associated with online surveys. These include the potential for the link to reach non-US medical schools through forwarding, excluding potential participants due to incorrect email addresses within a group’s lists, or self-selecting by forwarding through networks of students who have already volunteered to take the survey, which limits how random and representative the surveyed sample is. Online surveys are particularly prone to lower response rates, as was seen in this study’s response rate as well; however, those who complete the survey did so voluntarily, which is better for the quality of the responses. Research has also shown there can be differences in response rates between genders, as seen with a predominantly female response to this study. While it is common for women to respond to survey questionnaires in higher numbers than males, this is a limitation that needs to be acknowledged when extrapolating the results to the medical student population as a whole [17]. Using expected responses from males and females (based on enrollment figures for 2015–2016 from the American Association of Medical Colleges: 48 % female and 53.2 % male) [18] and the observed rates of 74.3 % female and 25.2 % male in our dataset, weighted values were produced. Responses form the attitudes and perceptions categories fell slightly in agreement; however, responses on exposures to sex and gender topics increased overall.

This study was limited by a binary (male and female) representation of gender. It is important in the future to study the health outcomes seen in the transgender population and evaluate ways in which gender health across the continuum can be better addressed in curriculum.

### Next steps

Based on results of this survey, the next step is to delineate effective integration of SGBM knowledge into mainstream medical education curriculum. Sex and gender influences can be found across the entire health spectrum from birth to death, therefore, threading concepts of sex and gender across the student’s 4-year learning experience through the integration of evidence-based sex and gender knowledge. This approach might avoid the large financial outlays and stagnation that can occur with stand-alone curricula and avoid requests for large blocks of curriculum time and resources which require resources to be subtracted from other curricular areas. Many would agree that finding time for new content in today’s packed curriculum is challenging and viewed less favorably by curriculum leaders. Thus, the threading concept could help engage faculty to participate in the process of sex and gender curriculum integration and garner buy-in from curriculum gate keepers [19].

**Fig. 2:**
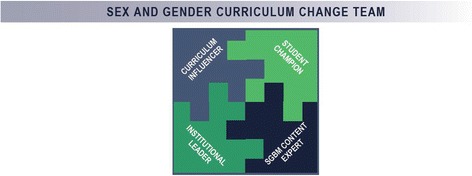
Sex and Gender Medical Education Curriculum Change Team

Effective curricular implementation demands strong institutional leadership. One institution’s integration of sex and gender-based medicine led to the creation of a “change team” with four key members (Fig. 2). This model for curricular change is outlined in the Sex and Gender Medical Education Summit Proceedings available at http://sgbmeducationsummit.com/. The “Curriculum Influencer” with the help of “SGBM Content Experts” develops and delivers curricular content with clear articulation of sex and gender principles.

Along with identifying faculty leaders who will lead integration of evidenced-based sex and/or gender differences, it is important to delineate core competencies in sex and gender medicine [20]. These objectives and competencies can be presented to students through integrative techniques. A curriculum mapping approach can link areas of SGBM knowledge from didactic studies to clinical competencies that are weaved throughout existing curriculum.

The inconsistency of reported exposure to certain sex- and gender-specific content areas throughout medical school curriculum from year one to year four indicates a need for more cohesive integration of gender-specific principles [21]. Faculty development and support from an “Institutional Leader” is crucial to systematically incorporate SGBM principles into medical content areas from first year to fourth year. Lastly, it is imperative to utilize the “Student Champion(s)” member of the change team to not only engage learners but create the future generation of SGBM institutional leaders.

## Conclusions

This survey not only identified knowledge gaps, but also highlighted the dramatic awareness of students in regards to sex and gender differences and their desire to know more. Integration across UME is the goal and will require not only curricular transformation but an inherent culture change from the current assumption that not knowing the difference means there is no difference. Linking the concepts of sex and gender medicine to precision or individualized medicine may help students and institutions further clarify the relevance of such principles. We must assist current health professionals in recognizing the increasing body of knowledge around sex and gender differences, and even more so, passing this knowledge to future providers. The results of this unique and landmark survey should encourage ongoing commitment of US medical school leadership, professional organizations, learners, accrediting bodies, and other stakeholders to close the sex and gender medicine gap in our current curricula.

## Additional file

Additional file 1: Sex and Gender Medical Education National Student Survey. (DOCX 330 kb)

## References

[1] Boston Women’s Health Book Collective (BWHBC) (1977). Our bodies, ourselves.

[2] Wizemann TM, Pardue ML, Institute of Medicine (US) Committee on Understanding the Biology of Sex and Gender Differences (2001). Exploring the biological contributions to human health: does sex matter?.

[3] Legato MJ (2004). Principles of gender-specific medicine.

[4] Regitz-Zagrosek V (2012). Sex and gender differences in health. EMBO Rep.

[5] Gender affects patient outcomes. 2014 AEM Consensus Conference. University of Cincinnati; n.d.

[6] Appleman Y (2015). Sex differences in cardiovascular risk factors and disease prevention. Atherosclerosis.

[7] Greenblatt DJ (2013). Gender differences in pharmacokinetics and pharmacodynamics of zolpidem following sublingual administration. J Clin Pharmacol.

[8] Food US, Administration D (2013). FDA drug safety communication: risk of next-morning impairment after use of insomnia drugs; FDA requires lower recommended doses for certain drugs containing zolpidem (ambien, ambien CR, edluar, and zolpimist).

[9] Council of graduated medical education (CoGME). Fifth Report: Women and Medicine. U.S. Department of Health and Human Services: Rockville, MD; 1995.

[10] Women’s Health in the Medical School Curriculum (1996). Report of a survey and recommendations.

[11] Henrich JB (2014). Women’s health education initiatives: why have they stalled?. Acad Med.

[12] Ashurst JV (2014). Emergency medicine gender-specific education. Acad Emerg Med.

[13] Miller VM, Rice M, Schiebinger L (2013). Embedding concepts of sex and gender health differences into medical curricula. J Women’s Health.

[14] Theobald J, Gaglani S, Haynes MR (2015). The association between confidence and accuracy among users of a mobile web platform for medical education. Ann Intern Med.

[15] Siriwardena AN, Irish B, Asghar ZB (2012). Comparing performance among male and female candidates in sex-specific clinical knowledge in the MRCGP. Br J Gen Pract.

[16] McGregor AJ (2014). Foundation for a novel emergency medicine subspecialty: sex, gender, and women’s health. Acad Emerg Med.

[17] Porter SR, Whitcomb ME (2005). Non-response in student surveys: the role of demographics, engagement and personality. Res High Educ.

[18] American College of Medical Associations. Table B-1.2: Total Enrollment by U.S. Medical School and Sex, 2011-2012 through 2015-2016. https://www.aamc.org/download/321526/data/factstableb1-2.pdf. Accessed 5 Jun 2016.

[19] Ludwig S, Oertelt-Prigione S, Kurmeyer C (2015). Successful strategy to integrate sex and gender medicine into a newly developed medical curriculum. J Women’s Health.

[20] Verdonk P, Mans LJL, Lagro-Janssen ALM (2005). Integrating gender into a basic medical curriculum. Med Educ.

[21] McGregor AJ (2013). Advancing sex and gender competency in medicine: sex and gender women’s health collaborative. Biol Sex Diff.

