# Supplementary material for: Sex and gender in medical education: a national student survey
Source: Biol Sex Differ. 2016 Oct 14;7(Suppl 1):45. doi: 10.1186/s13293-016-0094-6 (PMC5073801; doi:10.1186/s13293-016-0094-6)
Supplement: Additional file 1: — Sex and Gender Medical Education National Student Survey. (DOCX 330 kb) [file 13293_2016_94_MOESM1_ESM.docx]

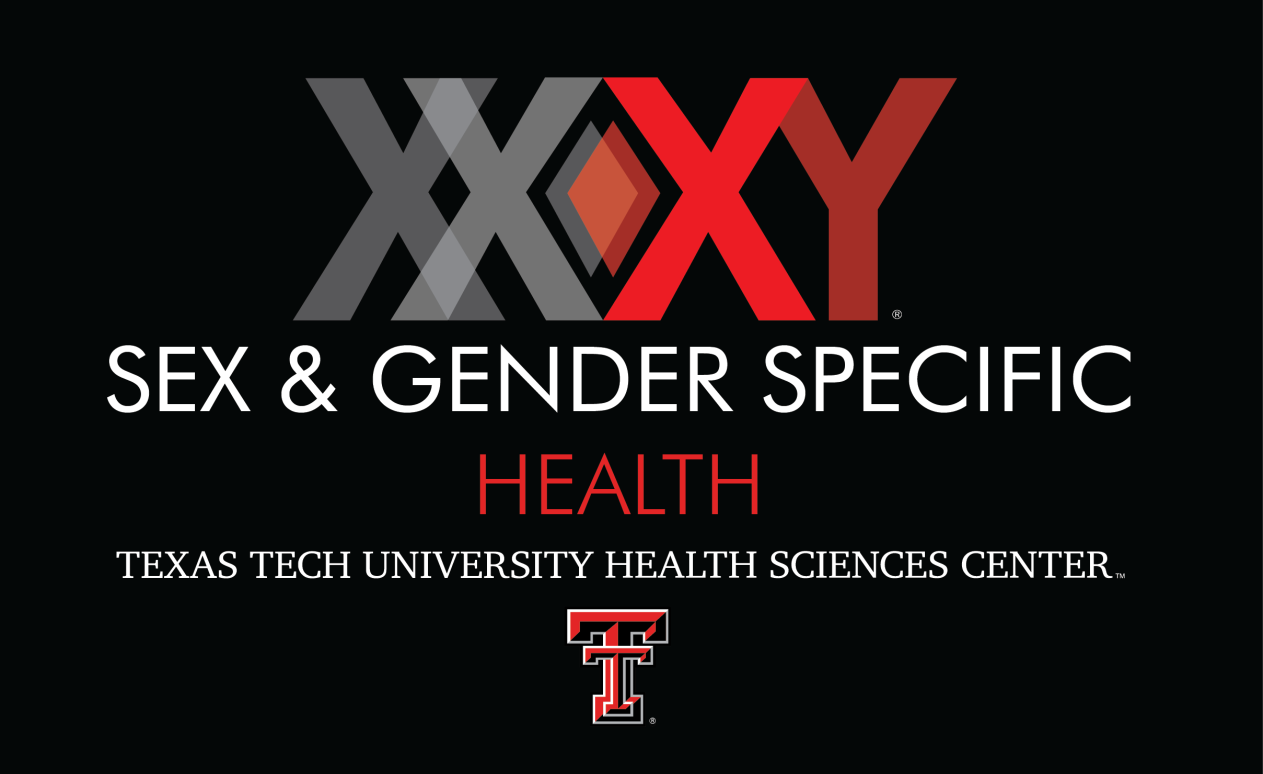


*Sex and Gender Medical Education National Student Survey*


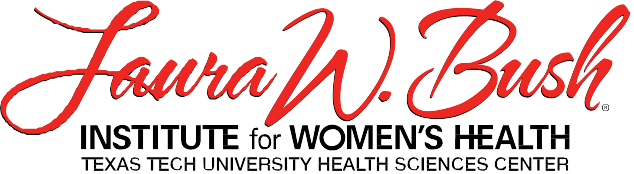


***Sex and Gender Medical Education National Student Survey***

Thank you for participating in this study. Over the course of the past 20 years, several initiatives have aimed at effectively integrating women’s health, women's health and gender specific medicine medical school curricula. However, the current status of these efforts has not recently been assessed. With a goal of facilitating the integration of such curricula we are undertaking a research project directed toward students enrolled in allopathic and osteopathic medical schools in the U.S., and their opinions on the extent to which their respective school addresses these topics.

This study is conducted under the Texas Tech University Health Sciences Center School of Medicine Institutional Review Board. Organizations supporting this survey effort include, the American Medical Student Association, the Asian Pacific American Medical Student Association, the American Medical Women’s Health Association, and the Student National Medical Association. Funding for this project is provided by the Laura W. Bush Institute for Women’s Health. By completing this confidential and anonymous instrument, you are giving your consent to participate in a research project. The survey will take less than 15 minutes to complete. Your participation is voluntary and you can stop at any time.

**Note:** For the purpose of completing this survey, please consider the following definitions of 'women's health', 'men's health', and ‘gender/sex specific medicine’ while completing the question sets:

***Women’s Health****: Screening, diagnosis and management of conditions that are unique to female anatomy, more prevalent in women, and/or more consequential in women*

***Men’s Health****: Screening, diagnosis and management of conditions that are unique to male anatomy, more prevalent in men, and/or more consequential in men*

***Sex and Gender-Specific Medicine****: The practice of medicine based on the understanding that biology (dictated by sex chromosomes) and social roles (gender) are important in and have implications for prevention, screening, diagnosis, and treatment; and in the design and implementation of health research, policy, programs and services in men and women.*

1 Please state the medical school that you attend: 2 In what state is your medical school located?

- Alabama (1)
- Alaska (2)
- Arizona (3)
- Arkansas (4)
- California (5)
- Colorado (6)
- Connecticut (7)
- Delaware (8)
- Florida (9)
- Georgia (10)
- Hawaii (11)
- Idaho (12)
- Illinois (13)
- Indiana (14)
- Iowa (15)
- Kansas (16)
- Kentucky (17)
- Louisiana (18)
- Maine (19)
- Maryland (20)
- Massachusetts (21)
- Michigan (22)
- Minnesota (23)
- Mississippi (24)
- Missouri (25)
- Montana (26)
- Nebraska (27)
- Nevada (28)
- New Hampshire (29)
- New Jersey (30)
- New Mexico (31)
- New York (32)
- North Carolina (33)
- North Dakota (34)
- Ohio (35)
- Oklahoma (36)
- Oregon (37)
- Pennsylvania (38)
- Rhode Island (39)
- South Carolina (40)
- South Dakota (41)
- Tennessee (42)
- Texas (43)
- Utah (44)
- Vermont (45)
- Virginia (46)
- Washington (47)
- West Virginia (48)
- Wisconsin (49)
- Wyoming (50)
- Other (51)

1. What is the approximate size of your entering first-year class of medical students? 3a What year are you in medical school?

- First year (1)
- Second year (2)
- Third year (3)
- Fourth year (4)
- Fifth year + (5)

3b What program are you enrolled in?

- MD only (1)
- DO only (2)
- MD/PhD (3)
- DO/PhD (4)
- MD with other combined degree program(s) (5)
- DO with other combined degree program(s) (6)
- Other (7)

3c What is your age?

- Less than 20 years (1)

🔾 21-25 years (2)

🔾 26-30 years (3)

🔾 31-35 years (4)

🔾 36-40 years (5)

🔾 41 + (6)

3d What is your gender?

- Man (1)
- Woman (2)
- Other (3)

3e Prior to medical school, what was your field(s) of study? (Check all that apply)

❑ Biological sciences (1)

❑ Physical sciences (2)

❑ Social sciences (3)

❑ Fine arts (4)

❑ Engineering (5)

❑ Business or Management (6)

❑ Healthcare studies (7)

❑ Women's studies (8)

❑ Other (9)

1. Please indicate which of the following describes the funding structure of your medical school:

- State-supported public institution (1)
- Private institution (2)
- Not sure (3)
- Other (4)

4a Please indicate which of the following describes the organizational structure of your medical school:

- Free-standing medical school (not affiliated with a larger Health Sciences Center) (1)
- Part of a multi-disciplinary university (that includes other health programs - e.g. Pharmacy, Nursing, etc.) (2)

4b What do you perceive to be the primary focus(es) of your medical school? (Check all that apply)

❑ Research (1)

❑ Clinical care (2)

❑ Medical education (3)

❑ Community engagement (4)

❑ Other (5)

1. Please indicate your level of agreement or disagreement with the following statements:

|  | | Disagree (2) | Neither Agree nor Disagree  (3) | Agree (4) | Strongly Agree (5) |
| --- | --- | --- | --- | --- | --- |
| I am familiar with the topic of sex and gender differences in medicine. (1) | 🔾 | 🔾 | 🔾 | 🔾 | 🔾 |
| Women's Health focuses solely on issues specific to females (menarche, pregnancy, menopause). (2) | 🔾 | 🔾 | 🔾 | 🔾 | 🔾 |
| Sex and gender medicine is the same as women's health. (3) | 🔾 | 🔾 | 🔾 | 🔾 | 🔾 |
| Knowing sex and gender differences improves one's ability to manage patients. (4) | 🔾 | 🔾 | 🔾 | 🔾 | 🔾 |
| The majority of medical knowledge is based on data obtained from males. (5) | 🔾 | 🔾 | 🔾 | 🔾 | 🔾 |
| Medical education | 🔾 | 🔾 | 🔾 | 🔾 | 🔾 |

| should include the teaching of sex and gender differences. (6)  My medical education has included the teaching of sex and gender differences. (7) | 🔾 | 🔾 | 🔾 | 🔾 | 🔾 |
| --- | --- | --- | --- | --- | --- |

1. Based on the following definition of "Women's Health," please answer the questions

below: Women's Health: Screening, diagnosis and management of conditions that are unique to female anatomy, more prevalent in women, and/or more consequential in womenDoes your medical school teach Women's Health outside of lectures and/or rotations on female-specific health issues?

- Yes (1)
- No (2)
- Not sure (3)

Answer If What year are you in medical school? Third year Is Selected Or What year are you in medical school? Fourth year Is Selected Or What year are you in medical school? Fifth year + Is Selected

6a

Answer If What year are you in medical school? Third year Is Selected Or What year are you in

medical school? Fourth year Is Selected Or What year are you in medical school? Fifth year +

Is Selected

|  | | No (2) | No, but I'd be interested in this (3) | Not sure (4) |
| --- | --- | --- | --- | --- |
| Does your institution have a Women's Health elective? (1) | 🔾 | 🔾 | 🔾 | 🔾 |
| Does your institution require readings from Women's Health textbooks? (2) | 🔾 | 🔾 | 🔾 | 🔾 |
| Does your institution offer a Women's Health fellowship? (3) | 🔾 | 🔾 | 🔾 | 🔾 |
| Does your institution offer a Women's Health residency program? (4) | 🔾 | 🔾 | 🔾 | 🔾 |
| Does your institution require Objective Structured Clinical Exams (OSCEs) or other simulated patient cases in Women's Health? (5) | 🔾 | 🔾 | 🔾 | 🔾 |
| Does your institution's library subscribe to Women's Health journals? (6) | 🔾 | 🔾 | 🔾 | 🔾 |
| Does your | 🔾 | 🔾 | 🔾 | 🔾 |

| institution's library subscribe to Gender- Specific Medicine journals? (7) |  |  |  |  |
| --- | --- | --- | --- | --- |

6b Does your medical school test medical students' knowledge of Women's Health?

- Yes (1)
- No (2)
- Not sure (3)

Answer If Does your institution test medical students' knowledge of Women's Health? Yes Is

Answer If Does your institution test medical students' knowledge of Women's Health? Yes Is Selected

6c Based on your current training year, in what years of your medical school curriculum has your knowledge of Women's Health been tested? (Choose all that apply.)

Selected

❑ First Year (1)

❑ Second Year (2)

❑ Third Year (3)

❑ Fourth Year (4)

6d Does your medical school assess medical students' attitudes toward Women's Health?

- Yes (1)
- No (2)
- Not sure (3)

6e Does your medical school offer student rotations in a Women's Health primary care clinic?

- Yes (1)
- No (2)
- Not sure (3)

6f Please provide any additional comments you have regarding your institution's curriculum and/or assessment of topics related to Women's Health.

1. Based on the following definition of "Men's Health," please answer the questions below: Men's Health: Screening, diagnosis and management of conditions that are unique to male anatomy, more prevalent in men, and/or more consequential in menDoes your medical school teach Men's Health outside of lectures and/or rotations on male-specific health issues (e.g. urology)?

- Yes (1)
- No (2)
- Not sure (3)

Answer If What year are you in medical school? Third year Is Selected Or What year are you in medical school? Fourth year Is Selected Or What year are you in medical school? Fifth year + Is Selected

7a

Answer If What year are you in medical school? Third year Is Selected Or What year are you in

medical school? Fourth year Is Selected Or What year are you in medical school? Fifth year +

Is Selected

|  | | No (2) | No, but I'd be interested in this (3) | Not sure (4) |
| --- | --- | --- | --- | --- |
| Does your institution have a Men's Health elective? (1) | 🔾 | 🔾 | 🔾 | 🔾 |
| Does your institution require readings from Men's Health textbooks? (2) | 🔾 | 🔾 | 🔾 | 🔾 |
| Does your institution offer a Men's Health fellowship? (3) | 🔾 | 🔾 | 🔾 | 🔾 |
| Does your institution offer a Men's Health residency program? (4) | 🔾 | 🔾 | 🔾 | 🔾 |
| Does your institution require Objective Structured Clinical Exams (OSCEs) or other simulated patient cases in Men's Health? (5) | 🔾 | 🔾 | 🔾 | 🔾 |
| Does your institution's library subscribe to Men's Health journals? (6) | 🔾 | 🔾 | 🔾 | 🔾 |

7b Does your medical school test medical students' knowledge of Men's Health?

- Yes (1)
- No (2)
- Not sure (3)

Answer If Does your medical school test medical students' knowledge of Men's Health? Yes Is

Answer If Does your medical school test medical students' knowledge of Men's Health?

Yes Is Selected

7c Based on your current training year, in what years of your medical school curriculum has your knowledge of Men's Health been tested? (Choose all that apply.)

Selected

❑ First Year (1)

❑ Second Year (2)

❑ Third Year (3)

❑ Fourth Year (4)

7d Does your medical school assess medical students' attitudes toward Men's Health?

- Yes (1)
- No (2)
- Not sure (3)

7f Please provide any additional comments you have regarding your institution's curriculum and/or assessment of topics related to Men's Health.

1. Based on the following definition of "Sex and Gender-Based Medicine" please complete the next question set. Sex and Gender-Based Medicine: The practice of medicine based on the understanding that biology (dictated by sex chromosomes) and social roles (gender) are important in and have implications for prevention, screening, diagnosis, and treatment; and in the design and implementation of health research, policy, programs and services in men and women.

8a Based on the definition above, does your institution have an identified Sex and Gender- Based Medicine curriculum?

- Yes (1)
- No (2)
- Not sure (3)

8b Does your curriculum include classes/programs on sex and gender differences?

- Yes (1)
- No (2)
- Not sure (3)

1. Please indicate your level of agreement with the following statements:

| Strongly Disagree (1) | | Disagree (2) | Neither Agree nor Disagree (3) | Agree (4) | Strongly Agree (5) |
| --- | --- | --- | --- | --- | --- |
| My curriculum has given me a better understanding of sex and gender medicine. (1) | 🔾 | 🔾 | 🔾 | 🔾 | 🔾 |
| My curriculum has prepared me to manage sex and gender differences in healthcare. (2) | 🔾 | 🔾 | 🔾 | 🔾 | 🔾 |

1. Sex and Gender-Specific Content Areas Please use the following definitions as a guide for your selection: 1= No courses or lectures2 = Minimal coverage o Defined as: A mention of sex specific differences in disease presentation during lecture 3 = Moderate coverage o Defined as: Male and female disease presentation is presented proportionately during lecture. 4 = Extensive coverage o Defined as: At least one complete lecture is dedicated to the way in which disease presentations vary between men and women. 5 = Don’t know For each of the following topics, please indicate the extent to which your institution covers sex and gender differences in your curriculum:

| 1 = No  Course or Lectures (1) | | 2 = Minimal  Coverage (2) | 3 = Moderate  Coverage (3) | 4 = Extensive  Coverage (4) | 5 = Don't  Know (5) |
| --- | --- | --- | --- | --- | --- |
| Medical history taking (1) | 🔾 | 🔾 | 🔾 | 🔾 | 🔾 |
| Domestic violence (2) | 🔾 | 🔾 | 🔾 | 🔾 | 🔾 |
| Substance abuse (3) | 🔾 | 🔾 | 🔾 | 🔾 | 🔾 |
| Mental health (4) | 🔾 | 🔾 | 🔾 | 🔾 | 🔾 |
| Nutrition (5) | 🔾 | 🔾 | 🔾 | 🔾 | 🔾 |
| Pharmacology (6) | 🔾 | 🔾 | 🔾 | 🔾 | 🔾 |
| Pulmonology (7) | 🔾 | 🔾 | 🔾 | 🔾 | 🔾 |
| Cardiology (8) | 🔾 | 🔾 | 🔾 | 🔾 | 🔾 |
| Rheumatology (9) | 🔾 | 🔾 | 🔾 | 🔾 | 🔾 |
| Infectious diseases (10) | 🔾 | 🔾 | 🔾 | 🔾 | 🔾 |
| Endocrinology (11) | 🔾 | 🔾 | 🔾 | 🔾 | 🔾 |

1. There are evidence-based health differences between men and women in regard to the topics listed below. Please answer yes or no regarding whether your medical education to date has included those differences in the following topics:

|  | Yes (1) | No (2) |
| --- | --- | --- |
| Presenting symptoms of myocardial infarction (1) | 🔾 | 🔾 |
| Using aspirin for the |  |  |
| prevention of myocardial | 🔾 | 🔾 |
| infarction and stroke (2) |  |  |
| Presenting symptoms of clinical depression (3) | 🔾 | 🔾 |
| Dosing of zolpidem (4) | 🔾 | 🔾 |
| Narcotic addiction (5) | 🔾 | 🔾 |
| Smoking cessation (6) | 🔾 | 🔾 |
| Sexual dysfunction (7) | 🔾 | 🔾 |
| Breast cancer (8) | 🔾 | 🔾 |
| Risk factors for the |  |  |
| development of osteoporosis | 🔾 | 🔾 |
| (9) |  |  |
| Outcomes after low impact fractures in adults (10) | 🔾 | 🔾 |
| Victims of domestic violence (11) | 🔾 | 🔾 |
| End-organ toxicity of alcohol use (12) | 🔾 | 🔾 |

1. Please provide any additional comments or questions you may have.
